# Taxonomic Affiliation of New Genomes Should Be Verified Using Average Nucleotide Identity and Multilocus Phylogenetic Analysis

**DOI:** 10.1128/genomeA.00927-14

**Published:** 2014-12-04

**Authors:** María José Figueras, Roxana Beaz-Hidalgo, Mohammad J. Hossain, Mark R. Liles

**Affiliations:** a Unitat de Microbiologia, Departament de Ciènces Médiques Bàsiques, Facultat de Medicina i Ciències de la Salut, IISPV, Universitat Rovira i Virgili, Reus, Spain; b Department of Biological Sciences, Auburn University, Auburn, Alabama, USA

## Abstract

The average nucleotide identity (ANI) determines if two genomes belong to the same species. Using ANI, we detected mislabeled genomes and recommend verifying with ANI and multilocus phylogenetic analysis the species affiliations of the announced genomes. The slightly different results obtained with different ANI calculation software can potentially mislead taxonomic inferences.

## COMMENTARY

The average nucleotide identity (ANI) is a similarity index between a given pair of genomes that can be applicable to prokaryotic organisms independently of their G+C content, and a cutoff score of >95% indicates that they belong to the same species (1, 2). Despite the fact that some of the genomes published in *Genome Announcements* include ANI values with closely related genomes (3–6), these data are missing in many studies. The genome sequencing studies may use different software packages for ANI determination (3–6). The currently available software tools for ANI calculation include programs that have to be downloaded, like JSpecies (http://www.imedea.uib.es/jspecies) and Gegenees (http://www.gegenees.org/documentation.html), as well as online calculation tools, like the one at the EzGenome (http://www.ezbiocloud.net/ezgenome/ani) and the ANI calculator (http://enve-omics.ce.gatech.edu/ani/index). However, there is a lack of information indicating whether these tools provide consistent results.

In a recent study using ANI calculations and a multilocus phylogenetic analysis (MLPA), we discovered that 14 (36%) of the 39 *Aeromonas* genomes deposited in the GenBank genome database were taxonomically mislabeled and that ANI values can vary using different calculation tools (7). Of the different tools employed, the ANI calculator always provided higher values than the JSpecies and the EzGenome tools that provided very similar results, but the magnitude of the differences depended on the specific genomes that were compared. For instance, the ANI calculator provided results between the genomes *Aeromonas hydrophila* HZM (GenBank accession no. JEMQ01) and *A. hydrophila* ATCC 7966^T^ (GenBank accession no. CP000462) of 89.0%, but when using JSpecies, the ANI was 86.6%. These results were <95% (the species ANI cutoff value) and therefore showed that the genome *A. hydrophila* HZM does not affiliate with *A. hydrophila*. This was also confirmed by the MLPA, because in the obtained tree, *A. hydrophila* HZM did not cluster with the type strain of *A. hydrophila* but with that of *Aeromonas caviae* instead. In fact, the ANI comparison between the genome of *A. hydrophila* HZM with that of *A. caviae* Ae398 (GenBank accession no. CACP01) showed results of >95% (98.4% with ANI calculator and 98.1% with JSpecies), clearly indicating that HZM belongs to the species *A. caviae.*

Typically, the differences between the ANI values obtained with the ANI calculator and JSpecies or EzGenome did not affect the species classification. The only exception might be a borderline ANI value (94.7%) obtained with the ANI calculator for the genomes of *Aeromonas veronii* AMC34 (GenBank accession no. AGWU01) and *A. veronii* B565 (GenBank accession no. CP002607) that might lead investigators to conclude that the two genomes belonged to the same species. However, the ANI values determined with JSpecies (93.7%) and EzGenome (93.5%), together with the MLPA results, confirmed that these two strains (AMC34 and B565) do not belong to the same species. Therefore, the use of other ANI determination methods in parallel with the ANI calculator is required to reinforce the correct interpretation of the results in borderline cases. Nevertheless, the ANI enabled us to easily detect wrongly labeled genomes.

The mistakes in the species names of deposited genomes are relevant because they may lead to incorrect conclusions in comparative genomic studies. Therefore, these data should motivate researchers to implement measures to prevent introducing taxonomical errors in public genome databases.

This commentary alerts authors about the ANI variations in relation to the calculation tool used and of the importance of using multiple approaches to confirm the taxonomic affiliations of announced genomes.
